# Image quality and radiation dose of cone-beam CT versus multidetector CT for upper extremity osteosynthesis

**DOI:** 10.1038/s41598-026-44687-y

**Published:** 2026-03-24

**Authors:** Aynur Gökduman, Scherwin Mahmoudi, Christian Booz, Philipp Reschke, Tommaso D’Angelo, Jennifer Gotta, Leona S. Alizadeh, Leon D. Grünewald, Simon Bernatz, Maral Nejati, Katrin Eichler, Thomas J. Vogl, Ibrahim Yel

**Affiliations:** 1 Clinic for Radiology and Nuclear Medicine, University Hospital Frankfurt, Frankfurt, Germany; 2 Diagnostic and Interventional Radiology Unit, Department of Biomedical Sciences and Morphological and Functional Imaging, “G. Martino” University Hospital Messina, Messina, Italy; 3 Department of Diagnostic and Interventional Radiology, Bundeswehrzentralkrankenhaus Koblenz, Koblenz, Germany

**Keywords:** Cone-beam computed tomography, Multidetector computed tomography, Image quality, Radiation dose, Anatomy, Diseases, Health care, Medical research, Oncology

## Abstract

This study compared image quality and radiation dose between cone-beam computed tomography (CBCT) and multidetector computed tomography (MDCT) under equivalent scanning settings, focusing on postoperative imaging after upper extremity osteosynthesis. A distal radius plate was implanted in a cadaveric forearm to simulate postoperative conditions. A total of twenty-four scans were performed using both modalities. Radiation dose was quantified with seven dosimeters placed at various anatomical locations and scan parameters were adjusted to ensure comparability. Subjective image quality was evaluated by five independent radiologists, while objective image quality was assessed using signal-to-noise ratio and contrast-to-noise ratio. Significant differences were found in radiation exposure and image quality. CBCT showed a slightly higher radiation dose (dose-length product: CBCT, 56.97 mGy*cm; MDCT, 46.19 mGy*cm; *p* < 0.0001). No significant difference was observed in cortical bone assessment (*p* = 0.28), but CBCT was rated higher for cancellous bone visualization (*p* = 0.005), artifact reduction (*p* = 0.003), and overall image quality (*p* = 0.009). Objectively, CBCT demonstrated lower image noise with superior signal-to-noise ratio (*p* = 0.0004) and contrast-to-noise ratio (*p* < 0.0001). This research offers a direct comparison of CBCT and MDCT using matched scan parameters on the same anatomical specimen, providing practical insights into image quality and radiation dose for optimizing postoperative orthopedic imaging protocols. CBCT demonstrates advantages in bone imaging and artifact management, while MDCT has superior potential for radiation dose reduction without compromising image quality.

## Introduction

Cone-beam computed tomography (CBCT) and multidetector computed tomography (MDCT) are established imaging modalities in musculoskeletal diagnostics. CBCT utilizes a cone-shaped X-ray beam and flat-panel detector to generate 3D images from a single rotation^1^. MDCT, in contrast, employs a spiral scanning technique with a narrow fan beam, enabling high-resolution imaging and excellent tissue contrast^2,3^. Both imaging techniques have important clinical applications. CBCT can be particularly useful when MDCT is not conveniently available, e.g. for certain interventional vascular, surgical or orthopedic imaging procedures^4,5^.

In orthopedic imaging of the extremities, especially for postoperative follow-up, CBCT has gained importance due to its compact size and lower cost^6,7^. Consequently, it assumes a significance comparable to that of MDCT in specific applications^8,9^. However, CBCT has limitations in soft tissue visualization and is more susceptible to metal artifacts^4^. Efforts to improve low-contrast resolution using a larger field of view (FOV) often lead to increased scan times and higher radiation exposure^10^.

MDCT remains the reference standard in emergency diagnostics and complex trauma due to its rapid and accurate visualization of traumatic lesions^11,12^, robust image quality, and advanced reconstruction techniques that effectively reduce radiation dose^13^. Nevertheless, MDCT also has its limitations, including sensitivity to metallic materials and the generation of artifacts that can affect image quality^14^.

Currently, few studies compare MDCT and CBCT in terms of image quality and radiation exposure after osteosynthesis for traumatic injuries^15–17^. Given their clinical relevance, evaluating image quality, dose-efficient protocols, and device performance is essential. Previous studies often lacked direct comparisons within the same patient population. Our study addressed this by using a single study group and included a phantom study to assess radiation exposure and image quality for both modalities in routine clinical use.

## Materials and methods

### Cadaver phantom

**Fig. 1:**
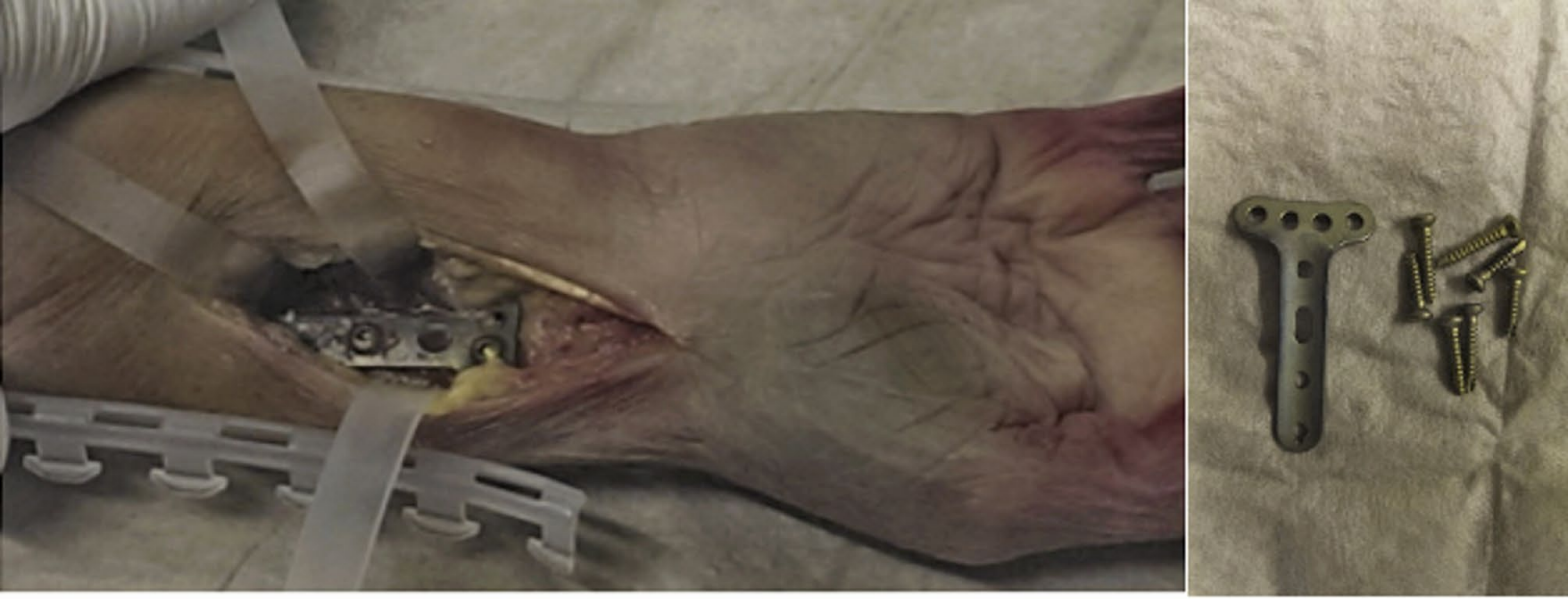
Photographic documentation of the freshly preserved cadaveric forearm after implantation of a distal radius plate. The left side of the image shows the surgical site view with the implanted plate at the distal radius. The right side displays the T-plate and the corresponding screw kit used for osteosynthesis. The image illustrates the anatomical alignment of the wrist in the palmar position and the positioning of the osteosynthesis material used to simulate postoperative imaging conditions for CBCT and MDCT.

To minimize patient radiation and allow comprehensive evaluation, a freshly preserved cadaveric forearm was used. A trauma surgeon implanted a distal radius plate (LCP-T, titanium/stainless steel) to simulate a postoperative scenario (Fig. 1), enabling standardized assessment of radiation exposure and image quality with both CBCT and MDCT.

### CBCT–examinations

CBCT scans were performed using the Carestream OnSight 3D Extremity System (Carestream Health, Rochester, NY, USA). Twelve scans were acquired with various combinations of tube voltage (90, 80, and 70 kV) and tube current-time products (5.0–2.0 mAs), starting with the manufacturer’s standard protocol (90 kV, 5.0 mAs). The selected tube voltage settings (70, 80, and 90 kV) represent the clinically available and manufacturer-recommended operating range of the CBCT system used in this study. Higher kV settings were not available on this device and therefore could not be included in the comparison. Each scan had an acquisition time of 25 s and used a cylindrical FOV measuring 22.3 × 22.3 × 22.3 cm^3^, incorporating a combination of 0.5-mm aluminum and 0.1-mm copper filters. Axial CBCT image datasets were generated with a slice thickness of 0.3 mm, corresponding to the smallest clinically available reconstruction setting of the system. In addition, coronal and sagittal multiplanar reformats were generated with a uniform slice thickness of 1.0 mm. The analysis focused exclusively on bony structures; soft tissue assessment was excluded due to system limitations.

*CBCT operator settings*.

**Table:** 

Parameters	Values			
kV	90		80	70
mAs	5.0; 4.1; 2.9; 2.0		5.0; 4.1; 2.9; 2.0	5.0; 4.1; 2.9; 2.0
FOV	22,3 × 22,3 × 22,3 cm^3^			
Slice thickness		Axial slice thickness: 0.3 mm, coronal and sagittal reformations: 1 mm each		
Scan time	25 s per scan			

### MDCT–examinations

MDCT scans were acquired with a SOMATOM Force dual-source CT system (Siemens Healthineers, Forchheim, Germany). To ensure comparability with CBCT, identical tube voltage settings were applied and scan parameters were configured in each system so that the resulting CTDI_VOL_ values were identical. MDCT imaging was performed with an axial slice thickness of 0.5 mm. Coronal and sagittal multiplanar views were generated at 1.0 mm, and all images were reconstructed using a bone-specific kernel (Q69) for standardized image evaluation. Post-processing was performed on syngo.via (VB10B, Siemens Healthineers).

*MDCT operator settings*.

**Table:** 

Parameters	Values		
kV	90	80	70
mA	68.0; 55.0; 40.0; 26.0	71.0; 59.0; 41.0; 28.0	70.0; 58.0; 41.0; 28.0
CT scan topogram measurement	25.6 cm		
Slice thickness	Axial slice thickness: 0.5 mm, coronal and sagittal reformations: 1 mm each		
Scan time	1.0 s		

### Dose calculation

**Fig. 2:**
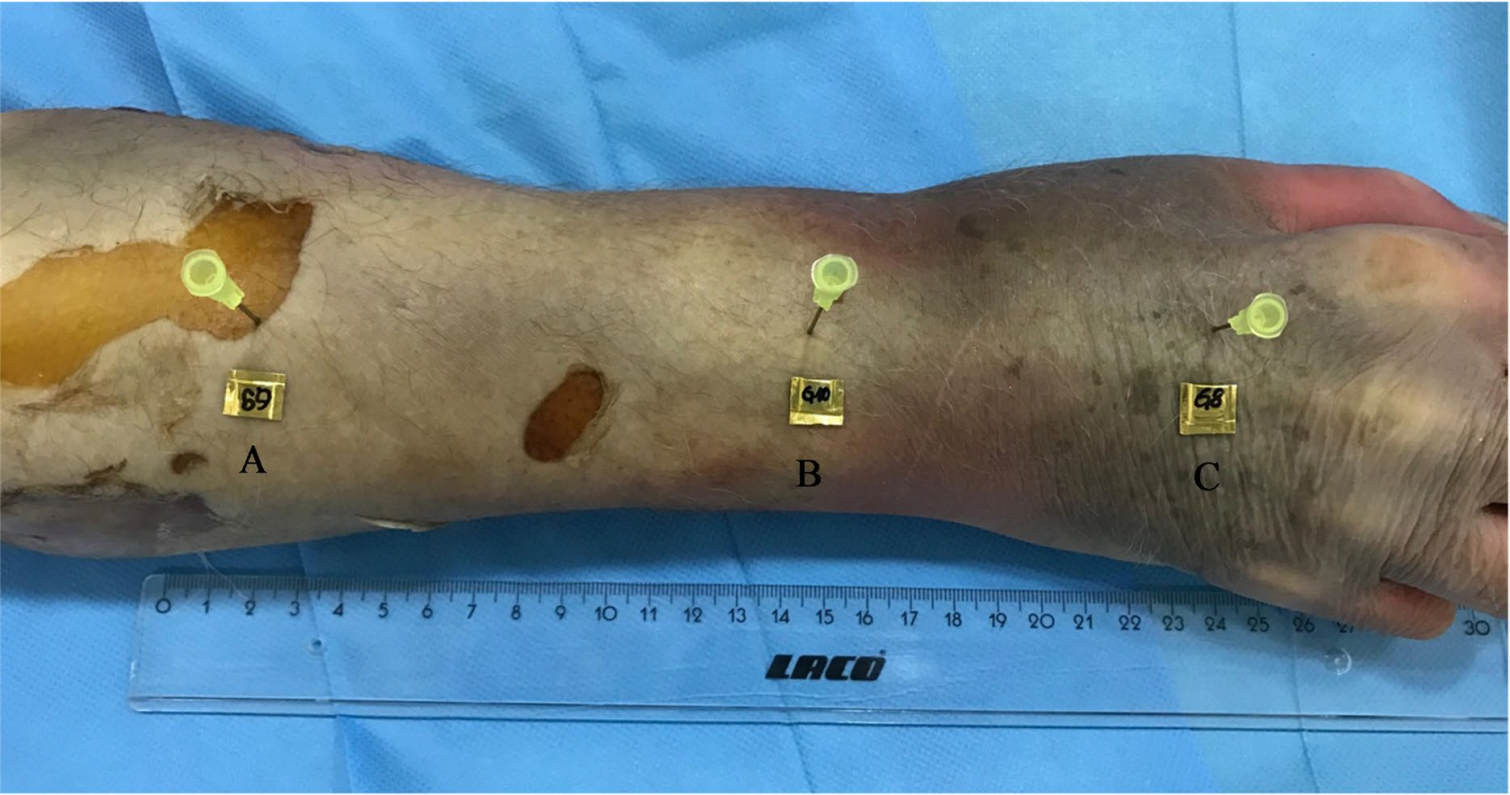
Photographic overview of the cadaveric forearm demonstrating the standardized placement of thermoluminescent dosimeters at predefined anatomical locations. Dosimeters were placed outside the scan field at a distance of approximately 30 cm from the scan volume (**A**), adjacent to the bone and implant (**B**), and on the skin surface (**C**) to enable assessment of equivalent and effective radiation doses during CBCT and MDCT imaging.

Seven dosimeters were placed at defined anatomical positions, three on the skin and bone surface, and one approximately 30 cm outside the scan area to measure radiation exposure, as illustrated in Fig. 2. Effective and equivalent doses were calculated. Radiation dose measurements were performed using thermoluminescent dosimeters (TLD). The TLDs were calibrated prior to the experiments according to the manufacturer’s specifications and used to assess equivalent and effective doses at predefined anatomical locations. The MDCT FOV was extended to match that of CBCT. Dose metrics were standardized using identical kV and CTDI_VOL_ values for both systems. CTDI_VOL_ is a standard dose metric for MDCT but is not directly applicable to CBCT due fundamental differences in beam geometry, acquisition technique, and detector design^17^. In this study, CTDI_VOL_ values were used as rough reference points for CBCT, achieved through adjustment of kV settings, to enable a balanced and pragmatic comparison of imaging protocols. An independent institute analyzed radiation exposure data from both MDCT and CBCT to ensure accurate and unbiased equivalent and effective dose determination.

### Subjective image quality

Five radiologists (1–15 years of experience) assessed image quality independently in a blinded setup. Using standardized monitors, they rated cortical bone, cancellous bone, artifacts, and overall quality on a 5-point Likert scale. In addition to the evaluation of individual image quality criteria (cortical bone, cancellous bone, metal-related artifacts), readers were asked to provide an independent rating of overall image quality. This global assessment reflected the reader’s overall diagnostic impression of the image and was not calculated as a composite or average of the individual criteria. Each image quality criterion was provided with ratings ranging from “moderate” to “excellent”. For each criterion, the average rating across all readers was subsequently calculated.

### Objective image quality

**Fig. 3:**
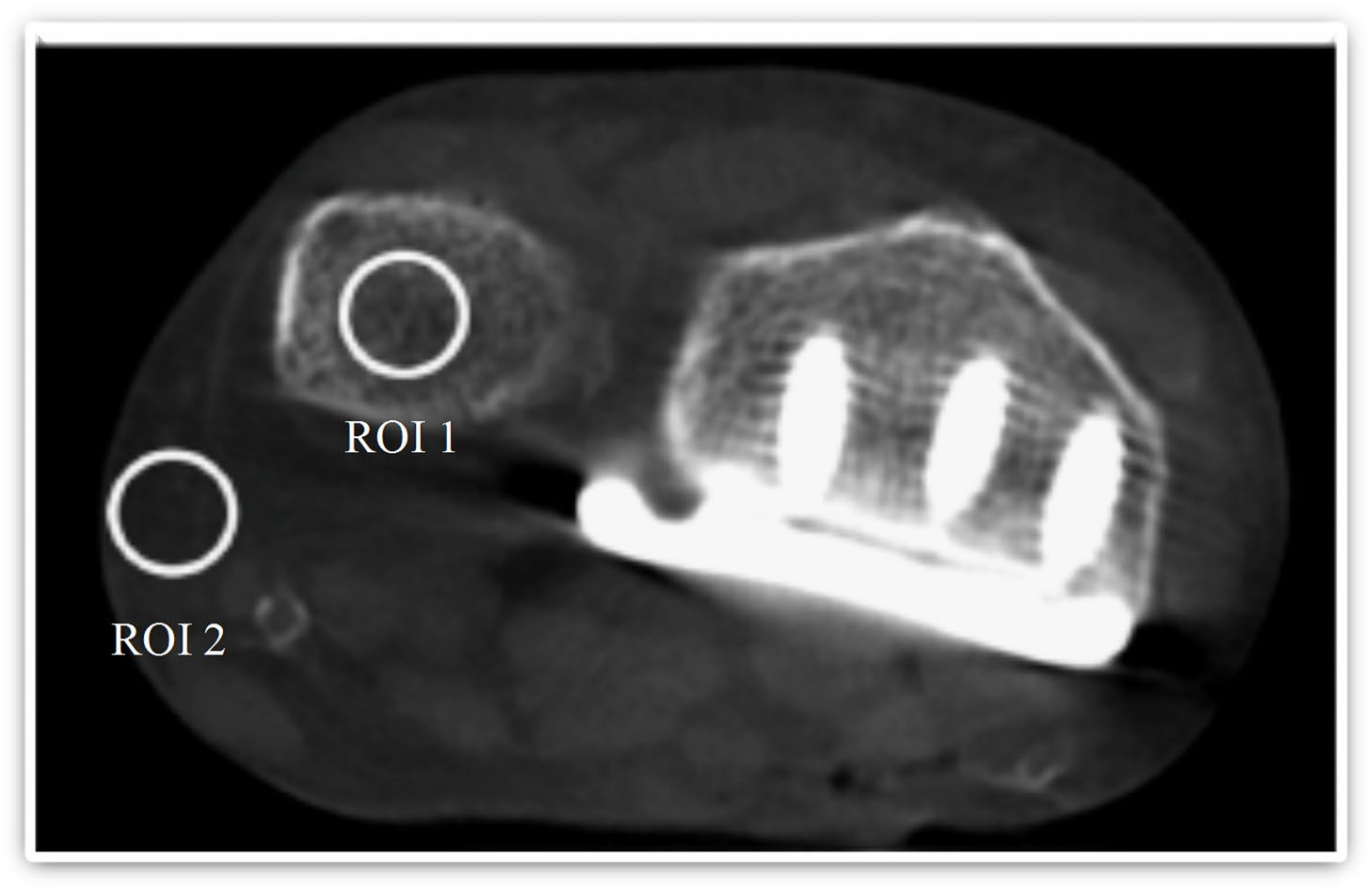
Representative axial image illustrating standardized regions of interest placed in trabecular bone (ROI 1) and subcutaneous fat (ROI 2) for objective image quality assessment. Regions of interest were defined to calculate signal-to-noise and contrast-to-noise ratios.

Objective image analysis was performed on the distal ulna following placement of the radius plate. Signal-to-noise ratio (SNR) and contrast-to-noise ratio (CNR) were calculated using Hounsfield unit (HU) measurements from trabecular bone and subcutaneous fat. Regions of interest (ROIs) were consistently positioned according to a standardized protocol based on I. Yel, C. Booz, M. H. Albrecht et al.^18^, as demonstrated in Fig. 3. Each measurement was taken twice and averaged, with care taken to avoid artifacts. SNR and CNR were calculated using the following formulas^19,20^:

$$\mathrm{S}\mathrm{N}\mathrm{R}=\frac{HU\left(ulna\right)}{SD\left(fat\right)}$$

$$CNR=\frac{HU\left(ulna\right)-HU\left(fat\right)}{SD\left(fat\right)}$$

### Statistical analysis

Data were analyzed using GraphPad Prism version 8.0 (GraphPad Software, La Jolla, CA, USA; https://www.graphpad.com/) and IBM SPSS Statistics version 29.1.0 (IBM Corp., Armonk, NY, USA; https://www.ibm.com/products/spss-statistics). Normality was assessed with Shapiro-Wilk and Kolmogorov-Smirnov tests. Depending on data distribution, t-tests or Mann-Whitney U-tests were applied.

Subjective image quality scores derived from Likert scales were treated as ordinal data and compared between modalities using the Mann–Whitney U test.

Objective image quality parameters, including signal-to-noise ratio (SNR) and contrast-to-noise ratio (CNR), were treated as continuous variables. Depending on data distribution, comparisons between modalities were performed using either unpaired t-tests or Mann–Whitney U tests.

A p-value of ≤ 0.05 was considered statistically significant.

## Results

### Radiation exposure

#### Dose-length product (DLP)

All examinations were performed using standardized CTDI_VOL_ and kilovolt peak (kV) parameters to ensure consistent image quality assessment. However, differences in dose-length products (DLPs) between MDCT and CBCT were observed, as detailed in Table 1. Across all scan protocols, CBCT demonstrated a slightly higher dose overall. The mean DLP was significantly higher in CBCT (57.0 mGy*cm) compared to MDCT (46.2 mGy*cm), with a statistically significant difference (*p* < 0.001).

**Table 1:** Dosimetric parameters.

MDCT			CBCT			
Scan setting (kV/mA)	CTDI_VOL_ (mGy)	DLP (mGy x cm)	Scan setting (kV/mAs)	CTDI_VOL_ (mGy)	DLP (mGy x cm)	
90/68	4.4	91.0	90/5.0	4.46	111.9	
90/55	3.56	73.5	90/4.1	3.66	91.8	
90/40	2.53	52.3	90/2.9	2.59	64.9	
90/26	1.64	34.5	90/2.0	1.79	44.8	
80/71	3.16	66.4	80/5.0	3.24	81.4	
80/59	2.65	55.7	80/4.1	2.66	66.7	
80/41	1.8	37.9	80/2.9	1.88	47.2	
80/28	1.24	26.1	80/2.0	1.30	32.6	
70/70	1.97	41.5	70/5.0	2.02	50.9	
70/58	1.65	34.7	70/4.1	1.66	41.7	
70/41	1.15	24.1	70/2.9	1.17	29.5	
70/28	0.79	16.6	70/2.0	0.81	20.3	
Mean value		46.2 ± 22.2 (24-68.4)			57.0 ± 27.3 (29.7–84.3)	*p* < 0.0001

#### Effective and equivalent dose

The results in Table 2 indicate that both the equivalent and effective doses were consistently higher for CBCT compared to MDCT. The mean effective dose was 0.0061 mSv for CBCT and 0.0048 mSv for MDCT (*p* < 0.0001). Although equivalent doses to the skin, bone surface, and out-of-field regions were slightly higher with CBCT, these differences were not statistically significant.

**Table 2:** Effective dose and equivalent doses of the different tissue types in MDCT.

Scan settings (kV/mA)	Skin (mSv)	Bone surface (mSv)	Out of area (mSv)	Effective dose (mSv)
90/68	0.001	0.0030	0.0068	0.0096
90/55	0.0001	0.0011	0.0009	0.0078
90/40	0.0007	0.0025	0.0043	0.0055
90/26	0.0005	0.0043	0.0032	0.0035
80/71	0.0009	0.0022	0.0052	0.0069
80/59	0.0007	0.0020	0.0044	0.0057
80/41	0.0006	0.0032	0.0033	0.0039
80/28	0.0004	0.0042	0.0026	0.0026
70/70	0.0006	0.0033	0.0035	0.0042
70/58	0.0005	0.0037	0.0030	0.0035
70/41	0.0004	0.0036	0.0024	0.0024
70/28	0.0001	0,0000	0.0008	0.0016

Effective dose and equivalent doses of the different tissue types in CBCT				
Scan settings (kV/mAs)	Skin (mSv)	Bone surface (mSv)	Out of area (mSv)	Effective dose (mSv)
90/5	0.0020	0.0040	0.0080	0.0115
90/4.1	0.0015	0.0024	0.0066	0.0095
90/2.9	0.0010	0.0041	0.0049	0.0069
90/2.0	0.0005	0.0013	0.0034	0.0049
80/5	0.0013	0.0029	0.0059	0.0085
80/4.1	0.0010	0.0030	0.0051	0.0070
80/2.9	0.0005	0.0018	0.0036	0.0051
80/2.0	0.0002	0.0015	0.0027	0.0037
70/5	0.0006	0.0033	0.0039	0.0055
70/4.1	0.0005	0.0031	0.0034	0.0046
70/2.9	0.0002	0.0022	0.0026	0.0034
70/2.0	0.0000	0.0000	0.0020	0.0025

Effective dose and equivalent doses of the different tissue types: Mean values ± SD (range)				
	MDCT	CBCT	*p*-value	
Skin (mSv)	0.0006 ± 0.0003 (0.0003–0.0009)	0.0008 ± 0.0006 (0.0002–0.0014)	0.114	
Bone surface (mSv)	0.0013 ± 0.0009 (0.0004–0.0022)	0.0019 ± 0.0012 (0.0007–0.0031)	0.076	
Out of area (mSV)	0.0033 ± 0.0017 (0.0016–0.005)	0.0043 ± 0.0018 (0.0025–0.0061)	0.052	
Effective dose (mSv)	0.0048 ± 0.0003 (0.0045–0.0051)	0.0061 ± 0.0027 (0.0034–0.0088)	< 0.0001	

### Subjective image quality

**Fig. 4:**
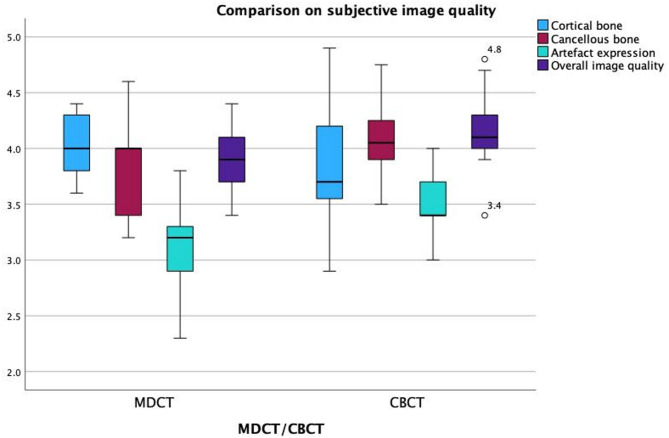
Boxplot comparison of subjective image quality ratings between CBCT and MDCT. Subjective ratings were performed by five blinded radiologists evaluating cortical bone, cancellous bone, artifacts, and overall image quality on a Likert scale.

Subjective image quality assessments were performed by five independent radiologists using a 5-point Likert scale. CBCT performed better than MDCT on many factors, as shown in Table 3; Fig. 4.

Cortical bone visualization showed no significant difference between the modalities (mean scores: CBCT 3.9, MDCT 4.0; *p* = 0.28). In contrast, CBCT was rated significantly better for visualizing cancellous bone (CBCT 4.12, MDCT 3.83; *p* = 0.006), reducing metal-related artifacts (*p* = 0.0032), and achieving higher overall image quality (CBCT 4.14, MDCT 3.90; *p* = 0.01). The only exception was under standard protocol settings, where both modalities received equal artifact ratings.

**Table 3:** Descriptive comparison of subjective image quality.

MDCT					CBCT				
Scan settings (kV/mA)	Subjective image quality				Scan settings (kV/mA)	Subjective image quality			
	Cortical bone	Cancellous bone	Artefact expression	Overall image quality		Cortical bone	Cancellous bone	Artefact expression	Overall image quality
90/68	4.4	4.6	3.8	4.4	90/5	4.85	4.75	3.8	4.8
90/55	4.2	4.0	3.4	4.0	90/4.1	4.2	4.3	3.8	4.2
90/40	4.4	4.0	3.7	4.2	90/2.9	4.9	4.7	3.6	4.7
90/26	4.0	4.0	3.2	4.0	90/2.0	4.2	4.0	3.4	4.0
80/71	3.8	3.4	3.2	3.8	80/5	3.8	4.2	3.4	4.4
80/59	4.4	4.2	3.2	4.0	80/4.1	3.8	4.2	3.6	4.1
80/41	4.0	4.0	3.2	3.8	80/2.9	3.6	4.1	3.4	4.1
80/28	4.2	4.0	3.0	4.2	80/2.0	3.6	3.9	4.0	4.1
70/70	3.8	3.4	2.9	3.4	70/5	3.5	3.9	3.0	4.0
70/58	3.6	3.8	2.7	3.8	70/4.1	3.6	4.0	3.4	3.9
70/41	3.6	3.4	2.9	3.6	70/2.9	3.5	3.9	3.4	4.0
70/28	3.8	3.2	2.3	3.6	70/2.0	2.9	3.5	3.2	3.4

Statistical comparison of subjective image quality: Mean data ± SD (range)									
	MDCT	CBCT	*p*-value						
Cortical bone	4.0 ± 0.3 (3.7–4.3)	3.9 ± 0.6 (3.3–4.5)	0.2841						
Cancellous bone	3.8 ± 0.4 (3.4–4.2)	4.1 ± 0.3 (3.8–4.5)	0.0059						
Artefact expression	3.1 ± 0.4 (2.7–3.5)	3.5 ± 0.3 (3.2–3.8)	0.0032						
Overall image quality	3.9 ± 0.3 (3.6–4.2)	4.1 ± 0.4 (3.8–4.5)	0.0097						

### Objective image quality

**Fig. 5:**
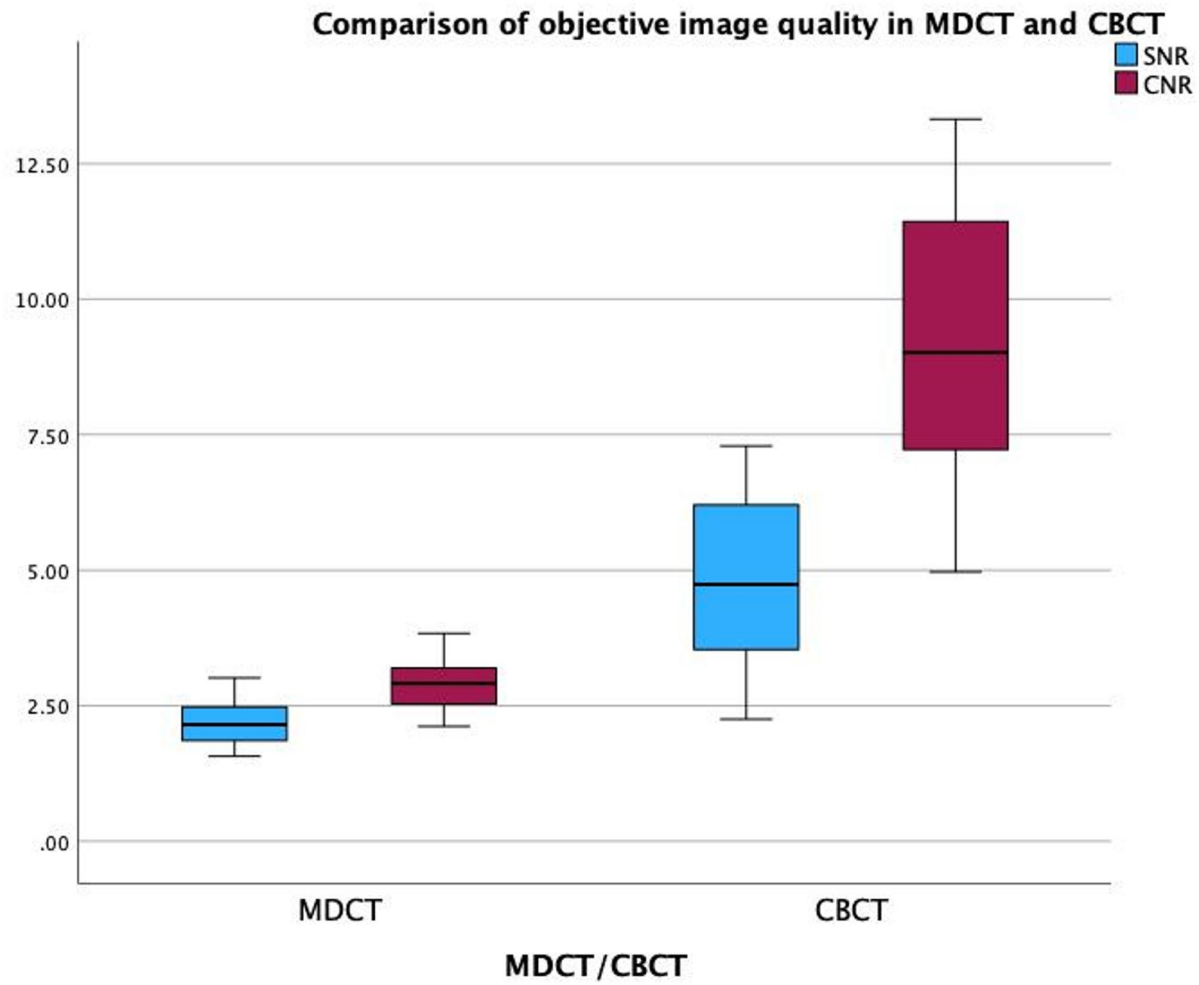
Boxplot comparison of objective image quality metrics between CBCT and MDCT. Signal-to-noise ratio (SNR) and contrast-to-noise ratio (CNR) were calculated from defined ROIs in trabecular bone and subcutaneous fat.

CBCT demonstrated significantly better performance in terms of image noise compared to MDCT (Fig. 5). The differences in signal-to-noise ratio (SNR, *p* = 0.0004) and contrast-to-noise ratio (CNR, *p* < 0.0001) were statistically significant. The SNR and CNR values for both imaging modalities are detailed in the subsequent Table 4.

**Fig. 6:**
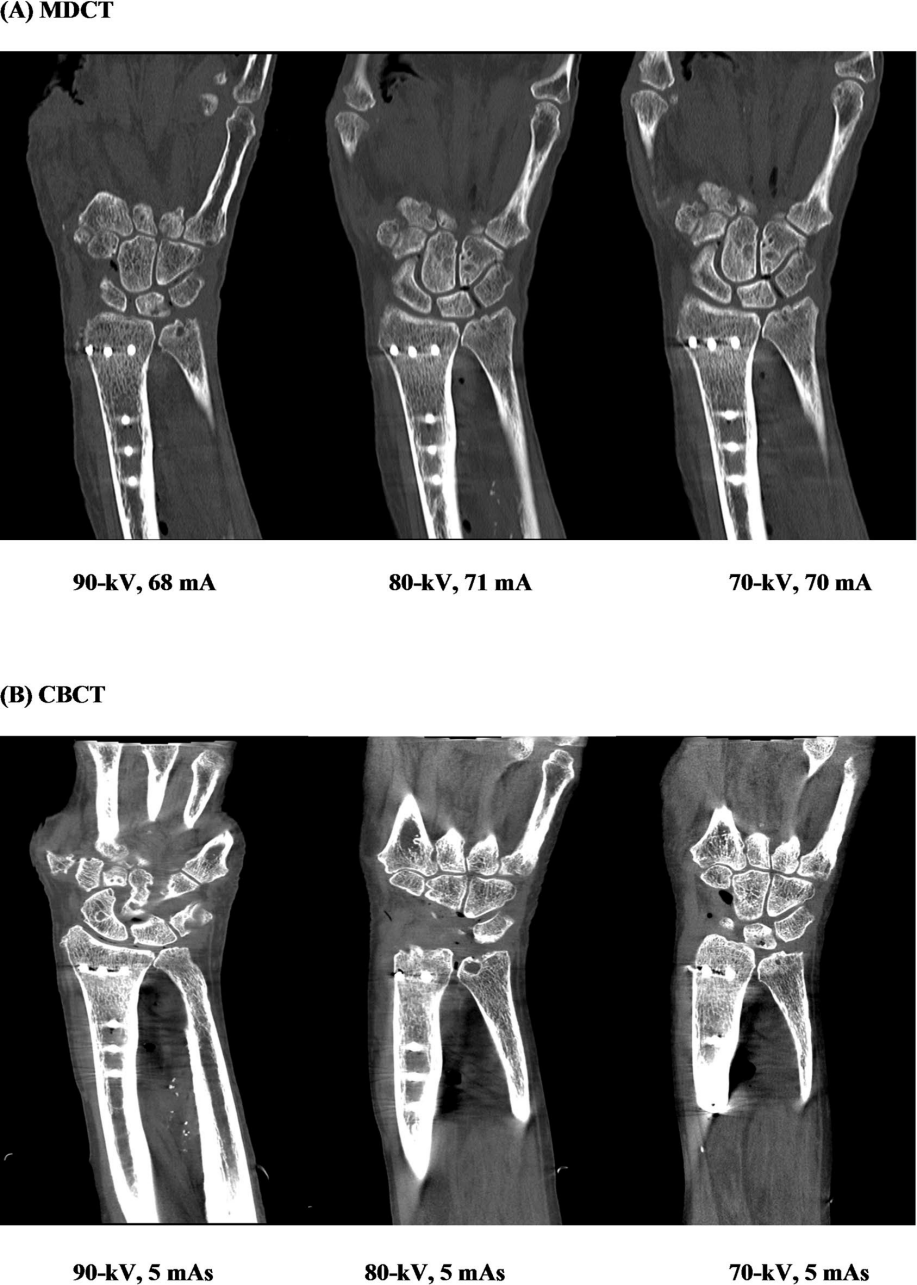
Representative MDCT (**A**) and CBCT (**B**) images acquired using different tube voltage and tube current settings. Images illustrate differences in image quality and metal-related artifact expression in the presence of a distal radius plate.

Figure 6 illustrates a visual comparison between MDCT (A) and CBCT (B) images acquired at different kV and mA/mAs settings, highlighting the impact of scan parameters on image quality and diagnostic clarity.

**Table 4:** Descriptive comparison of objective image quality.

MDCT			CBCT		
Scan settings (kV/mA)	SNR	CNR	Scan settings (kV/mAs)	SNR	CNR
90/68	2.30	3.13	90/5	4.31	7.76
90/55	2.11	2.89	90/4.1	6.50	10.64
90/40	1.59	2.38	90/2.9	7.29	13.32
90/26	2.05	2.79	90/2.0	5.90	9.77
80/71	3.02	3.84	80/5	6.62	12.82
80/59	2.07	2.69	80/4.1	5.18	10.83
80/41	2.38	3.13	80/2.9	3.68	6.53
80/28	1.66	2.22	80/2.0	3.40	7.09
70/70	2.77	3.57	70/5	2.25	4.97
70/58	2.57	3.27	70/4.1	3.73	8.26
70/41	2.2	2.93	70/2.9	5.44	12.04
70/28	1.57	2.12	70/2.0	2.51	7.35

Statistical comparison of objective image quality: Mean data ± SD (range)					
	MDCT	CBCT	*p*-value		
SNR	2.19 ± 0.46 (1.74–2.65)	4.73 ± 1.66 (3.07–6.40)	0.0004		
CNR	2.91 ± 0.52 (2.39–3.43)	9.28 ± 2.68 (6.60-11.96)	< 0.0001		

## Discussion

High-resolution imaging is essential for fracture detection, treatment planning and follow-up of traumatic injuries. In clinical routine, selecting an appropriate imaging modality requires a careful balance between image quality and radiation exposure. While CBCT and MDCT are both well-established in orthopedic imaging, direct comparisons are rare and often based on non-identical cohorts^21^. This study addresses that gap by evaluating both modalities under standardized, matched conditions using the same anatomical specimen with osteosynthesis material.

The results indicate that DLP values were comparatively lower with MDCT. However, in our study, the MDCT FOV had to be adjusted and extended to match that of CBCT in order to enable a direct comparison, which may have affected the measured radiation exposure. In clinical practice, MDCT typically operates at higher kV settings^22^. Additionally, MDCT offers a wider range of advanced protocol options, such as tin filtration, dual-energy CT (DECT), and metal artifact reduction (MAR), allowing for greater flexibility in scan parameter optimization. A study published in *European Radiology Experimental* demonstrated that the combination of tin filtration and high tube voltage significantly reduces metal artifacts in musculoskeletal CT imaging, thereby enhancing image quality. This combination enables more precise adaptation of scanning parameters to specific clinical needs^23^; however, these advanced techniques could not be incorporated into the present comparison.

The differences in radiation exposure in terms of dose equivalent for the skin, bone surface and skin outside of scanning area were not statistically significant, while the effective dose showed a significant superiority of MDCT over CBCT. This result is consistent with individual studies in the literature supporting the assumption that MDCT scans have a lower total radiation exposure than CBCT examinations. For example, J. Neubauer, C. Neubauer, A. Gerstmaier et al. showed that MDCT in hand imaging has good overall image quality with lower radiation dose and better dose optimization compared to CBCT^24^. Some studies in the existing literature even suggest that MDCT offers a lower absorbed dose with better image quality^25^. Although the effective dose was statistically higher for CBCT compared to MDCT, the absolute dose values for both modalities were very low. The measured effective doses (approximately 5–6 µSv) are substantially lower than those associated with a standard chest radiograph (approximately 100 µSv) and several orders of magnitude below the average annual natural background radiation (approximately 3 mSv)^26^. Therefore, while statistically significant, the observed dose difference is unlikely to be clinically relevant and is not expected to be a decisive factor when selecting an imaging modality for extremity examinations.

The CBCT used in our study is optimized for imaging bone structures and does not support soft tissue reconstruction, which limits the assessment of soft tissue structures. This limitation has also been identified in previous research^27^, as evidenced by the findings of M. Marx, F. Risi, B. Escudé et al.^28^, which suggests that CBCT provides excellent image quality for bone visualization but is less effective for soft tissue assessment. In scenarios where soft tissue structures need to be included in the imaging process, a longer scan time is often required to achieve optimal image quality. However, this increase carries a higher risk of radiation exposure, which must be carefully considered in clinical and research settings^29^. A study published compared the image quality and radiation dose CBCT and MDCT in pediatric extremity trauma. The findings indicated that MDCT provides superior image quality for soft tissue evaluation and is less affected by artifacts compared to CBCT. The authors concluded that while CBCT offers advantages in specific scenarios, MDCT remains the preferred modality for comprehensive assessment in pediatric extremity trauma^21^.

Our study results confirm that both CBCT and MDCT can be recognized as high-quality imaging techniques with remarkable usability of the generated images. In particular, CBCT shows certain advantages, especially in the visualization of spongiosa and the reduction of artifacts in the presence of metal implants. A significant advantage of CBCT over MDCT was observed in terms of SNR and CNR in postoperative scenarios involving metal implants. Several studies have shown that compared to MDCT, CBCT offers high spatial resolution with fewer metallic artifacts^29–31^. The superior SNR and CNR of CBCT enable the generation of clear, high-contrast images, even in challenging scenarios with metal structures. These features are particularly important for post-operative imaging, where precise visualization of anatomical structures surrounding the implants is crucial for assessing implant positioning, detecting potential complications and long-term monitoring^32^. However, the superiority of CBCT over MDCT in assessing bony structures was only partially confirmed, as no significant difference was found in cortical bone evaluation.

This study has several limitations that should be acknowledged. Only a single human cadaveric forearm specimen was used, which may limit the generalizability of the results. The use of a freshly preserved specimen represents a methodological strength, as tissue attenuation properties more closely resemble in vivo conditions compared with embalmed cadavers^33^. However, cadaver-based imaging cannot fully reproduce physiological conditions encountered in clinical practice. In particular, the absence of tissue perfusion and physiological motion, as well as potential post-mortem tissue changes, may influence image quality and radiation dose characteristics^27,34^. The standardized use of a single anatomical specimen enabled a controlled comparison between CBCT and MDCT under identical imaging conditions. Nevertheless, this approach does not capture interindividual variability in anatomy, soft tissue composition, body habitus, or patient size, all of which are known to substantially affect radiation dose and image quality in clinical CT imaging^35^. In addition, clinically relevant motion artifacts were not evaluated in this study. Furthermore, the analysis focused exclusively on osseous structures, and soft tissue evaluation and related diagnostic performance were not investigated. Finally, the results are specific to the CBCT and MDCT systems used in this study and may not be directly transferable to other devices.

Based on our findings, both CBCT and MDCT offer distinct advantages depending on clinical context and patient-specific factors. CBCT showed superior visualization of cancellous bone and better artifact reduction near metallic implants, making it particularly suitable for postoperative follow-up of small joints, especially in outpatient or surgical settings with limited MDCT access. The open gantry design of CBCT also allows greater flexibility during interventional procedures. While MDCT struggles with metal-induced artifacts, CBCT may perform better in such cases—though at the cost of reduced tomographic 3D resolution^36^. In contrast, MDCT remains the preferred modality for soft tissue imaging, including muscles, tendons, and ligaments, and for use in obese patients due to its broader field-of-view and greater penetration depth. Its faster acquisition times and protocol flexibility also make it ideal for emergency or complex trauma cases. In summary, CBCT serves as a valuable alternative for bone-focused imaging, particularly in the presence of metal implants, while MDCT remains essential for comprehensive diagnostics involving soft tissue and wider anatomical coverage.

## Data Availability

The datasets used and analyzed during the current study are available from the corresponding author upon reasonable request.

## Abbreviations

CBCT: Cone-beam computed tomography
CNR: Contrast-to-noise ratio
CTDI_VOL_: Computed tomography dose index
DECT: Dual-energy computed tomography
DLP: Dose-length product
FOV: Field of view
HU: Hounsfield units
IT: Iterative reconstruction
kV: Kilovoltage peak
mA: Milliampere
mAs: Milliampere-seconds
MAR: Metal artifact post-processing
MDCT: Multidetector computed tomography
MSK: Musculoskeletal
ROI: Region-of-interest
SD: Standard deviations
SNR: Signal-to-noise ratio
